# Can the Tumor Microenvironment Alter Ion Channels? Unraveling Their Role in Cancer

**DOI:** 10.3390/cancers17071244

**Published:** 2025-04-06

**Authors:** Rosaria Gentile, Davide Feudi, Luana Sallicandro, Andrea Biagini

**Affiliations:** 1 Department of Chemistry, Biology and Biotechnologies, University of Perugia, Via dell’Elce di Sotto 8, 06123 Perugia, Italy; rosaria.gentile@dottorandi.unipg.it; 2 Department of Biostatistics, Epidemiology and Public Health, University of Padua, Via L. Loredan 18, 35131 Padova, Italy; davide.feudi@studenti.unipd.it; 3 Department of Medicine and Surgery, Perugia Medical School, University of Perugia, Piazza Lucio Severi 1, 06132 Perugia, Italy

**Keywords:** tumor microenvironment, TRPM5, K_ATP_, connexins, pannexins, ion channels

## Abstract

Neoplastic cells are characterized by metabolic reprogramming, known as the Warburg effect, in which glucose metabolism is predominantly directed toward aerobic glycolysis, with reduced mitochondrial oxidative phosphorylation and increased lactate production even in the presence of oxygen. This phenomenon provides cancer cells with a proliferative advantage, allowing them to rapidly produce energy (in the form of ATP) and generate metabolic intermediates necessary for the biosynthesis of macromolecules essential for cell growth. It is important to understand the role of ion channels in the tumor context since they participate in various physiological processes and in the regulation of the tumor microenvironment. These changes may contribute to the development and transformation of cancer cells, as well as affect the communication between cells and the surrounding microenvironment, including impaired or altered expression and functionality of ion channels. Therefore, the aim of this review is to elucidate the impact of the tumor microenvironment on the electrical properties of the cellular membranes in several cancers as a possible therapeutic target.

## Simple Summary

The tumor microenvironment (TME) plays a crucial role in regulating ion channel activity, influencing cancer growth and progression. This review examines the involvement of K_ATP_, TRPM5, and gap junctions (connexins and pannexins) in tumors, highlighting their impact on cellular mechanisms and communication. It explores how acidic pH, extracellular ATP, and lactate within the TME modulate these channels, influencing tumor cell survival. Finally, therapeutic strategies targeting ion channels are discussed as novel approaches for cancer treatment.

## 1. The Tumor Microenvironment: A Complex Cancer Ecosystem

Tumor cells have an extraordinarily high demand for nutrients to sustain their anabolic needs and energy production rates. However, unlike normal cells, cancer cells exhibit greater metabolic plasticity, allowing them to better adapt to lower or changing nutrient conditions [1]. Consequently, the concept of the local microenvironment playing a crucial role in regulating cell behavior has become increasingly accepted in cancer biology [2,3].

Mechanistic studies, including those in preclinical tumor models, have demonstrated that tumor microenvironment (TME) cells and their secreted molecules are pivotal in cancer pathogenesis, making them attractive therapeutic targets [4]. Furthermore, recent research has highlighted the importance of non-cellular components of the niche, particularly the extracellular matrix (ECM), in cancer progression [5,6,7,8]. Although long considered a stable structure that primarily supports tissue morphology, the ECM is now recognized as a dynamic and versatile component of the cellular environment that influences fundamental aspects of cell biology [9]; through direct and indirect mechanisms, the ECM regulates nearly all cellular behaviors and it is indispensable for major developmental processes [3,10,11,12].

Cancer cells orchestrate a tumor-supportive environment by recruiting and reprogramming non-cancerous host cells and by remodeling the vasculature and ECM [13]. This dynamic process relies on heterotypic interactions between cancer cells and resident or recruited non-cancerous cells within the TME [13]. Recent advances in computational analysis and modeling using single-cell transcriptomic data, bulk tumor expression profiles, and spatial transcriptomics [14,15,16] have revealed a diversity of intercellular signaling networks within the TME. These atlases serve as powerful hypothesis-generating datasets that guide functional studies, helping to elucidate how complex intercellular interactions contribute to the formation and evolution of the TME. Various mechanisms regulate this intercellular dialogue, including direct cell–cell contact and paracrine signaling [17]. One of the key factors shaping the tumor microenvironment is its altered ionic and metabolic landscape, which significantly influences cancer progression. A notable feature is the acidic nature of the TME, which has been documented across many types of cancer for several decades [18]. The extracellular pH (pH_e_) in cancer tissue can reach as low as 6.5, representing a roughly 10-fold increase in free H^+^ concentration compared to normal tissue, and it can vary spatially and temporally between a value of 6.5 and 7.6 [18,19,20,21]. This shift in pH_e_ is one of the most pronounced ion concentration disturbances in the body, influencing the behavior of cancer cells and their interactions with surrounding normal and immune cells [22]. To maintain intracellular pH (pH_i_) homeostasis and support survival in this hostile environment, cancer cells rely on specialized ion transporters and channels. Among these, Na^+^/H^+^ exchangers (NHEs) exert an important role in maintaining pH_i_ by extruding H^+^ ions in exchange for extracellular Na^+^ [23,24,25]. Their activity supports cancer cell survival under acidic conditions by preventing excessive intracellular acidification. Additionally, NHEs contribute to tumor cell migration and invasiveness by generating local pH gradients that modulate cell–matrix interactions and cytoskeletal dynamics [26,27,28]. Given their role in tumor progression, inhibition of NHEs has been explored as a potential therapeutic strategy, with studies showing that targeting these transporters can reduce proliferation, enhance programmed cell death [24,29], and improve responses to chemotherapy [30,31]. Net elimination of intracellular acid occurs through transporters that either move H^+^ directly across the plasma membrane or transfer acid–base equivalents such as HCO_3_^−^ or lactate, which are in equilibrium with H^+^ in the cytoplasm and extracellular space [32,33,34,35,36]. In cancer tissue, pH_i_ is often equal to or higher than pH_e_, and due to the inside-negative membrane potential, H^+^ elimination from cancer cells requires energy input via primary or secondary active transporters, which have significant implications for tumor growth [35,37].

Breast cancer cells, for example, exhibit greater resistance to intracellular acidification compared to normal breast epithelial cells [32]. However, the low pH_e_ in solid tumors means that the pH_i_ of cancer cells can still be lower than that of normal cells in non-acidic environments. Under extreme extracellular acidification, cancer cells may reach a critically low pH_i_ that impairs metabolism, proliferation, and survival [35]. Beyond pH regulation, the ionic composition of the interstitial fluid in solid tumors differs significantly from that of corresponding normal tissues. Additionally, the interstitial concentration of K^+^ can reach up to 30 mM [38,39], while extracellular adenosine triphosphate (ATP) levels can rise to the hundreds of micromoles [40].

Accelerated glycolysis, a hallmark of cancer cells, generates intermediates for anabolic processes and provides ATP for energy-requiring cellular activities [41]. This phenomenon, known as the Warburg effect, occurs even in the presence of oxygen, where cancer cells rely on glycolysis rather than oxidative phosphorylation (OXPHOS) to generate ATP. Despite the raised ATP demand, intracellular ATP concentrations are typically elevated in cancer cells; however, disruptions in ATP production can induce cancer cell death and counteract acquired drug resistance [42,43,44]. This metabolic shift is accompanied by increased lactate production, as lactate is typically secreted by fermentative cancer cells, and accumulates in the tumor microenvironment [45,46]. Lactate, produced from pyruvate via fermentation, helps maintain glycolysis by replenishing NAD^+^ levels [47,48,49]. This excess lactate can be taken up and metabolized by oxidative cells in the tumor or elsewhere in the body, though recent evidence suggests that lactate may also be utilized within the fermentative cancer cells themselves [50]. It has also been proposed that lactate production, or lactogenesis, would be at the foundation of carcinogenesis and the main reason for the occurrence of the Warburg effect even in normoxia, due to its function as “lactormone” in cell signaling [51].

Increased lactate concentrations in tumors are typically higher than in plasma and normal tissue [32,46,52,53,54], and hyperpolarized 13C magnetic resonance spectroscopic imaging reveals pronounced lactate accumulation in tumors with reduced perfusion [55]. Measurements of lactate in the tumor microenvironment are sometimes lower than in plasma, indicating metabolic heterogeneity within tumors [56]. Studies utilizing microdialysis report that interstitial lactate concentrations in tumors are significantly elevated compared to adjacent normal tissues, such as in astrocytomas [57] and head and neck cancers [58]. In murine models, lactate concentrations in tumor tissues are four- to five-fold higher compared to those in normal tissue, while glucose concentrations are significantly reduced [32,46], further supporting the idea that lactate accumulates and glucose is depleted in the tumor microenvironment.

These dramatic deviations in the chemical environment have profound functional consequences, potentially influencing disease progression, therapeutic efficacy, and the development of treatment resistance [22].

## 2. Ion Channels and Cancer

Ion channels can be regulated by external stimuli such as voltage changes [59], ligand binding [60], or mechanical modifications [61]. Ion channels can be strongly affected by the tumorigenic process, where malignant cells modify their function to promote uncontrolled proliferation, survival, and invasion. The modification of the TME, including changes in ion concentrations, leads to opening and closing of the gates, affecting physiological processes such as cell communication, proliferation, and secretion [62,63,64].

**Figure 1:**
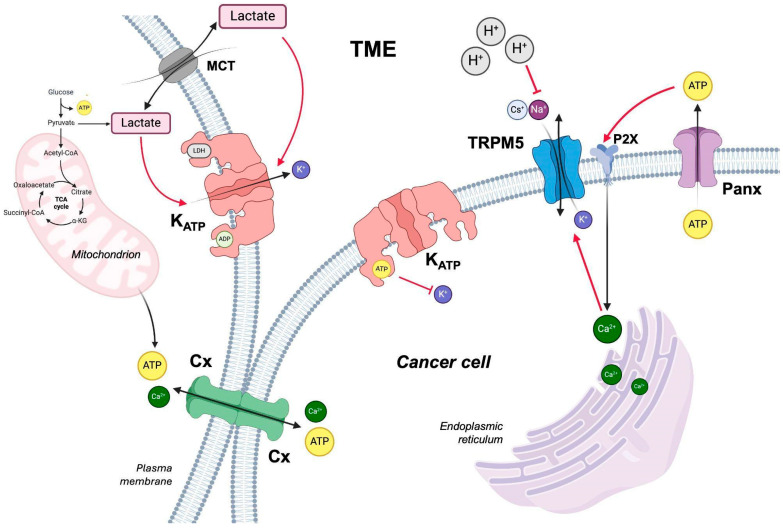
Crosstalk and regulation of ion channels in the tumor microenvironment (TME). This schematic illustrates the interactions of various ion channels in the tumor microenvironment (TME) of a cancer cell. Connexin (Cx) facilitates ATP transfer between adjacent cells, promoting intercellular communication. The K_ATP_ channel is inhibited by ATP but activated by intracellular and extracellular lactate. Pannexin (Panx) mediates ATP release into the extracellular space, where ATP binds to the purinergic receptor P2X, triggering an increase in intracellular Ca^2+^ levels, stored in the endoplasmic reticulum. Elevated Ca^2+^ concentration activates TRPM5, but its activity is suppressed by protons in the acidic TME. Lactate, transported via monocarboxylate transporters (MCT), contributes to microenvironmental modulation, influencing ion homeostasis, signaling, and tumor progression. Red arrows indicate the activation, red T bars indicate inhibition of the channels, and black arrows indicate the passage of the molecules through the channels in the extracellular and intracellular matrix. Image created with BioRender.com (accessed on 11 March 2025).

This review focuses on some of the ion channels identified to date to be involved (Figure 1), directly or indirectly, in tumorigenesis and/or tumor progression, which is closely related to the modification of the surrounding tumor microenvironment. In this context, the channels act as communicators between the latter and healthy and tumor cells (Table 1). Therefore, it is important to fully understand their mechanism.

### 2.1. ATP-Sensitive Potassium Channels in Cancer

ATP-sensitive potassium channels (K_ATP_) are widely expressed in the living organism [65,66,67,68,69], where they regulate various biological functions. The involvement of K_ATP_ in cancer pathophysiology is increasingly recognized, owing to their ability to link cellular metabolism with ionic homeostasis and membrane electrical activity [70]. K_ATP_ channels are located both in the plasma membrane and mitochondria [71,72], and can contribute to cancer cell growth, survival, and progression through different mechanisms [73].

K_ATP_ is included in the inward rectifying potassium channel (K_ir_) superfamily because of its electrophysiological characteristics. These channels are composed of a hetero-octameric complex consisting of four K_ir_6.x subunits (K_ir_6.1 or K_ir_6.2), which form the channel pore, and four regulatory sulfonylurea receptor (SUR_x_) subunits (SUR1, SUR2A, or SUR2B) [74,75,76].

The gating of these channels is based on a complex regulatory mechanism that allows them to respond to changes in the cellular energy state. Their activity is primarily determined by the intracellular ratio of adenosine nucleotides, ATP/ADP, which exerts both inhibitory and stimulatory effects. An increase in the ATP/ADP ratio closes K_ATP_ channels (leading to depolarization), while a decrease in the ATP/ADP ratio opens K_ATP_ channels (leading to hyperpolarization) [76,77]. In particular, ATP, by directly binding to the K_ir_6.x subunits, inhibits channel opening, while ADP, especially in the presence of Mg^2+^ ions, binds to the SUR_x_ subunits, stimulating channel opening [78].

In the tumor microenvironment, the low ATP/ADP ratio generated by glycolysis activates K_ATP_ channels, contributing to the setting of the resting membrane potential and promoting cell survival under metabolic stress conditions [79].

When K_ATP_ channels activate, they facilitate K^+^ efflux, keeping the membrane polarized and generating the electrochemical gradient necessary for Ca^2+^, a second messenger, to enter the cell through calcium release-activated channels (CRAC) and transient receptor potential canonical (TRPC) on the membrane [80]. Ca^2+^ is involved in various signaling pathways, including those that induce cell proliferation and mitosis [81], thereby contributing to disease progression.

The relationship between the aberrant expression of the ATP binding cassette subfamily C member 8 (ABCC8) and ABCC9 subunits, which encode for SUR1 and SUR2 [82], respectively, the overexpression of K_ATP_ channels, and the invasive growth of various malignant tumors, including liver, pancreatic, gastric, brain, bladder, and prostate cancers, has been confirmed by multiple studies [83,84,85].

In glioma cells [85,86], the opening of K_ATP_ channels has been associated with the activation of the extracellular signal-regulated kinase 1/2/mitogen-activated protein kinase (ERK/MAPK) signaling pathway, which regulates cell cycle progression and survival. Pharmacological inhibition of these channels with glibenclamide or diazoxide has been shown to block cell growth, arresting the cycle at the G1/S phase and increasing apoptosis [84]. A role for these channels has also been identified in human papilloma virus (HPV) pathogenesis. In cervical cancer, upregulation of the channel induced by the E7 oncoprotein has been reported, leading to tumor progression through the MAPK/activator protein-1 (AP-1) pathway. The same authors also reported that K_ATP_ channels induce an increase in oncoprotein expression via a positive feedback mechanism [87].

Using direct electrophysiological measurements of K_ATP_ channel activity in pancreatic β-cells and α-cells, both in humans and mice, it has been observed that a glycolytic metabolome locally controls K_ATP_ channels on the plasma membrane [88]. K_ATP_ channels are not only regulated by mitochondrial ATP as previously thought, but also locally by glycolytic enzymes. Upper glycolysis enzymes (glucokinase and phosphofructokinase) produce ADP, which activates K_ATP_ channels, while lower glycolysis enzymes (pyruvate kinase) use ADP to produce ATP, which closes K_ATP_ channels [89]. Glycolytic enzymes form a functional complex near the plasma membrane, facilitating substrate channeling, that is, the direct transfer of metabolites between consecutive enzymes. These enzymes are not randomly distributed in the cytoplasm but are directly anchored to the plasma membrane near K_ATP_ channels. This allows for the creation of metabolic microcompartments, ensuring a localized and efficient ATP production that directly regulates K_ATP_ channels without relying on mitochondrial ATP [88].

Cancer cells, like the pancreatic β-cells described by Ho and colleagues [88], primarily rely on glycolytic ATP for local cellular functions without depending on mitochondrial oxidation. This suggests that K_ATP_ channels in cancer cells may be regulated by a similar glycolytic metabolon, making them sensitive to glucose availability.

Because of the Warburg effect and the predominant glycolytic metabolism even in the presence of oxygen, cancer cells produce high amounts of lactate as the final metabolite of glycolysis [90]. Beyond its role in NAD^+^ recycling, lactate can act as a true metabolic signal capable of modulating cellular excitability. Available evidence suggests that excess lactate exported into the extracellular space can re-enter the cell through monocarboxylate transporters (MCT1/MCT4), increasing the NAD^+^/NADH ratio and activating K_ATP_ channels [88,91]. In contrast, lactate produced directly by membrane-associated lactate dehydrogenase (M-LDH) does not appear to influence K_ATP_ channels, as it is immediately recycled in the NAD^+^/NADH cycle to support local glycolysis and ATP production.

It has been demonstrated that, in skeletal muscle cells and cardiac myocytes, M-LDH physically associates with sarcolemmal K_ATP_ channels [92]. Through immunoprecipitation and immunofluorescence techniques, the authors showed that M-LDH is part of a protein complex that regulates channel activity. Furthermore, the application of extracellular lactate, studied using an “inside-out patch-clamp” configuration, revealed an increase in K_ATP_ channel current density. The channel-induced membrane hyperpolarization reduced calcium influx and protected the cell from ischemic stress, even in the presence of high ATP concentrations [93].

The inhibition of M-LDH, achieved using oxamate (a competitive inhibitor that blocks the conversion of pyruvate to lactate) and the removal of the enzyme through ultracentrifugation and membrane fractionation, led to a reduction in K_ATP_ channel activity and decreased sensitivity to lactate, demonstrating that the protein is necessary for their normal functioning [92]. This distinction between exogenous and endogenous lactate suggests that lactate can function as a metabolic signaling molecule, playing a key role in regulating membrane potential and enhancing the metabolic resilience of cancer cells [94]. An acidic pH_e_, characteristic of the tumor microenvironment, enhances K_ATP_ channel activation by acting on two specific amino acids, Thr71 and His175 [95], contributing to cell survival under stress conditions [82,96].

Other endogenous modulators regulate the activity of K_ATP_ channels in tumor cells, such as phosphatidylinositol-4,5-bisphosphate (PIP_2_), which interacts with K_ir_6.x subunits, stabilizing the open channel and promoting potassium efflux and calcium entry, which are crucial for cell proliferation [97]. Long-chain acyl-CoAs (LCCoAs), derived from lipid metabolism, also stimulate channel opening, especially in lipid-rich tumor microenvironments, such as prostate and colon cancer [97,98].

The activity of K_ATP_ channels is also regulated by kinases such as protein kinase A (PKA), PKC, and AMP-activated protein kinase (AMPK). PKA, activated by increased cAMP levels, promotes channel opening [99,100], while PKC phosphorylates K_ir_6.2, modulating the channel depending on the specific isoform involved [101]. PKC activation, driven by increases in diacylglycerol (DAG) and Ca^2+^, is often linked to lipid metabolism dysregulation in tumors. Both mechanisms influence membrane potential and cell survival, promoting tumor progression [102]. AMPK modulates K_ATP_ channels by promoting their opening [103]. In the tumor context, AMPK plays a dual role: it can act as a tumor suppressor by downregulating the mechanistic target of rapamycin kinase (mTOR), thereby inhibiting cell growth, but it can also promote cancer cell survival in unfavorable environments [104].

The involvement of K_ATP_ channels in key signaling pathways makes them promising targets for the development of new anticancer therapies. However, further studies are required to fully elucidate their molecular functions in different cancer types and explore their potential as novel pharmacological targets.

### 2.2. TRPM5 in Cancer

The transient receptor potential (TRP) channel superfamily consists of 28 different TRP ion channels in mammals, permeable to a wide range of cations such as Ca^2+^, Mg^2+^, Cs^+^, Na^+^, and K^+^. These channels are classified into subfamilies determined by amino acid sequences homologies, including TRPA (ankyrin), TRPC (canonical), TRPM (melastatin), TRPML (mucolipin), TRPN (NO-mechano-potential, NOMP), TRPP (polycystin), and TRPV (vanilloid), which are mainly responsible for sensory responses after different stimuli such as heat, pressure, and pH [105]. Being involved in chronic pain, TRP channels have been extensively studied in the context of cancer pain, particularly in relation to bone pain caused by cancer metastasis [106]. This review will focus on TRPM5, which belongs to the TRPM subfamily consisting of eight members (TRPM1 to TRPM8), with specific expression patterns and ion selectivity. Dysfunction of TRPM channels is linked to several disorders. TRPM2 is associated with Alzheimer’s and Parkinson’s disease due to its role in oxidative stress response [107,108]. Mutations of TRPM6 and TRPM7 can lead to hypomagnesemia in cardiovascular disease [109], since they are crucial to maintaining Mg^2+^ homeostasis, whereas TRPM8 is a cold-sensitive channel related to chronic pain and cancer progression [110,111]. TRPM4 is involved in arrhythmias and stroke [112], while TRPM5 dysfunction affects insulin resistance, contributing to metabolic disorders [113,114].

Structurally, TRPM channels are transmembrane proteins that form functional tetramers. Each subunit contains a large cytosolic region, a transmembrane domain, and a C-terminal region. The cytosolic domain ranges from 732 to 1611 amino acids and includes regulatory sites that influence the channel’s activity. The transmembrane domain consists of six helices (S1–S6), with the pore formed by a loop between S5 and S6, allowing the passage of ions. The C-terminal region varies among TRPM family members but usually includes a coiled-coil domain, essential for oligomerization and functional regulation. A distinctive feature of the TRPM family is the N-terminal TRPM homology region (MHR), a conserved sequence present in all TRPM channels. This region is important in channel assemblage and its function. While the C-terminal domain differs among TRPM members, ensuring functional properties, the N-terminal domains allow a common framework within the family [115,116,117,118]. In particular, TRPM5 channels are activated by intracellular Ca^2+^ at concentrations of 0.3–1 μM, which open the ion-conducting pore by binding the specific site located in the intracellular domain [119], whilst it is inhibited at higher concentrations. Conformational changes in TRPM5 structure also occur when ligands bind on the antagonist site between the S1–S4 domains and the pore domain, resulting in a closed state of the channel [120].

TRPM channels are involved in different physiological functions, including temperature sensation, taste perception, Ca^2+^ and Mg^2+^ homeostasis, oxidative stress response, and neurotransmission [105]. Specifically, TRPM5 mediates signaling in taste and other chemosensory cells, and plays an essential role in insulin secretion [119].

Furthermore, TRPM channels may also be distinct biophysically, as among them only TRPM4 and TRPM5 are the only nonselective cation channels permeable to Na^+^, K^+^, and Cs^+^ ions, but Ca^2+^-impermeable [121], probably due to the negatively charged residue in the pore loop determining their permeabilization [122]. However, TRPM5 is activated by the increase in intracellular Ca^2+^ [123], as well as temperature [124], and its activity is negatively affected by acidic pH_e_, resulting in a rapid reversible block in current (IC_50_: pH = 6.2) and a slower irreversible inactivation of current [125]. Although up-to-date knowledge regarding the relationship between cancer and TRPM5 is still poor, it has been linked to metabolic changes in the TME, potentially influencing tumor progression in metabolic disorders like diabetes-associated cancers [113,114,126].

A study carried out using univariate and multivariate Cox regression analyses to identify and validate TRP gene signatures for colon adenocarcinoma (COAD) found a positive correlation between TRPM5 gene expression and the multi-drug resistance gene (MDR1), associated with a poor overall survival rate in the COAD patients with higher expressions [127]. TRPM5, along with TRPV4 and TRPML1, was found to represent a clinically independent prognostic variable for patients with COAD. This result was also observed in another study, where the overexpression of TRPM5 was positively correlated with worse prognosis of colorectal cancer, confirming its role as a risk predictor [128].

Within the TRP channel family, TRPM5 was the only one that showed a significant prognostic value in triple-negative breast cancer (TNBC), a type of breast cancer where estrogen receptor, progesterone receptor, and human epidermal growth factor receptor 2 (HEGF2) are all negatively expressed [129]. A higher mRNA and protein expressions were found also in vitro in MDA-MB-231 (highly aggressive, invasive, and poorly differentiated TNBC cell line) and MDA-MB-468 (basal-like tumors of TNBC and epithelial-like morphology) cell lines, compared to the non-tumorigenic epithelial cell line MCF-10A.

An in vitro study on mouse melanoma cells found that triphenylphosphine oxide (TPPO), a selective TRPM5 blocker, dose-dependently inhibited acidic pH_e_-induced matrix metalloproteinase-9 (MMP-9) production (IC_50_: 41 mM after 24 h), but did not reduce cell viability. TPPO significantly inhibited spontaneous lung metastasis, despite no correlations being found by immunohistochemical staining for TRPM5. In the same study, in silico analysis showed a significant correlation between high levels of TRPM5 expression and shorter overall survival in patients with melanoma and gastric cancer, but not with lung, breast, and rectum cancers. Trpm5 mRNA expression was also induced by acidic pH_e_, but it was not inhibited by TPPO treatment [130]. The same research group also demonstrated that LLCm1 cells responded to transient acidification with an increase in Trpm5 mRNA, but this result was not confirmed in LLCm1 acidic pH_e_-adapted (LLCm1A) cells, suggesting that chronic exposure to the acidification of the tumor microenvironment promotes an adaptation both in genotype and phenotype of cells [131].

Despite the lack of information regarding TRPM5 in cancer, its susceptibility to several stimuli such as pH_e_, temperature, and intracellular calcium, typical of the TME, makes it a possible target for cancer therapy.

### 2.3. Pannexins and Connexins in Cancer

Gap junctions (GJs) are largely known as large-pore channels capable of regulating the intracellular exchange of ions, second messengers, nutrients, and cellular metabolites [132,133]. This review will focus mainly on pannexins (Panx) and connexins (Cx), which seem to be involved in cancer progression and connected to TME [134].

**Figure 2:**
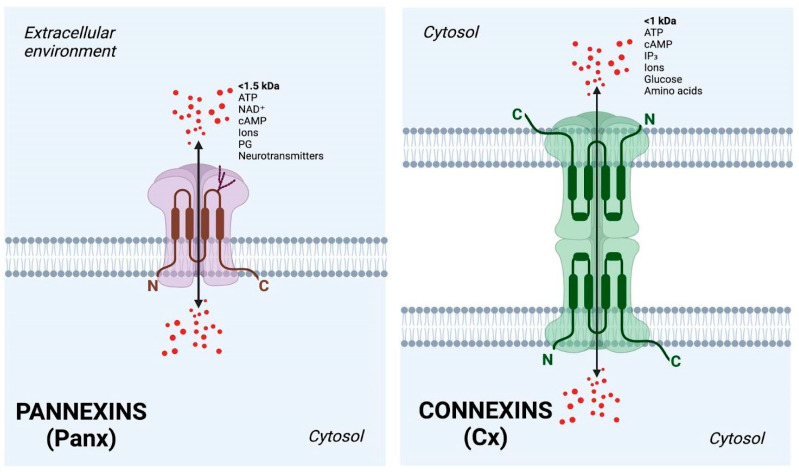
Comparison between the structures of pannexins (Panx) and connexins (Cx). Four transmembrane regions are linked by two extracellular loops and a single cytoplasmic loop with both the N- and C-terminal ends positioned within the cytosol. On the left, pannexins form hemichannels, containing two cysteine (Cys) loops, and one of them undergoes glycosylation (tree-like structure) and releases chemical signals (<1.5 kDa) into the extracellular environment, such as adenosine triphosphate (ATP), nicotinamide adenine dinucleotide (NAD^+^), adenosine 3′,5′-cyclic monophosphate (cAMP), ions (Ca^2+^, K^+^, Na^+^, Cl^−^), prostaglandins (PGs), and neurotransmitters such as gamma-aminobutyric acid (GABA) and glutamate. On the right, connexins undergo cell–cell interactions, containing three cysteine residues in each of their extracellular loops, allowing the direct exchange of small molecules (<1 kDa) such as glucose, second messengers like inositol 1,4,5-trisphosphate (IP₃), ATP, cAMP, ions (Ca^2+^, K^+^, Na^+^, Cl^−^), and small amino acids between adjacent cells. Image created with BioRender.com (accessed on 26 March 2025).

Pannexins are large-pore ion channels that allow the passage of larger molecules [135]. Along with connexins, pannexins have an important role as intracellular channels that allow metabolic and ionic coupling (Figure 2). They are also non-junctional hemichannels that work as paracrine signaling pathways by releasing ATP and modulating intracellular Ca^2+^ in astrocytes in culture [134]. Initial characterization in *Xenopus* oocytes supported the idea that pannexins can form gap junctions [136]; however, some evidence suggests that glycosylation at the extracellular domain prevents this channel family from undertaking cell–cell interaction [137]. The pannexin family is characterized by three different subtypes (Panx1–3). Panx1 and Panx2 are expressed in almost all tissues [138,139,140,141], while the expression of Panx3 is more restricted (skin, heart, osteoblast cells, and cartilage). All of them are involved in several pathophysiological events, such as pain, neurogeneration, epilepsy, inflammasome activation, and, particularly, Panx2 in diabetes. Structurally, these channels are formed in heptomers with seven-fold symmetry down the axis of the pore. The structure of each Panx1 protomer contains four transmembrane helices linked together by extracellular and intracellular domains and cytoplasmic-oriented N- and C-termini, and the transmembrane helices are similar to those of connexins [142].

In humans, 21 Cx genes are known to encode for gap junction proteins, which are expressed in all tissues except differentiated skeletal muscle, erythrocytes, and mature sperm cells. Stable noncovalent interactions between two hemichannels located in the plasma membranes of adjacent cells, through an H-bond between extracellular loops of their connexins, form a single GJ channel, also called a connexon. Gap junctions allow communication between adjacent cells, with connexins serving as their core proteins. The intracellular loop determines the specific properties of different connexins, such as conductance, pH dependence, voltage dependence, and selective permeability [143,144]. Cx are essential for communication between astrocytes to maintain the homeostasis of the central nervous system, which, however, can be compromised by pathogenic stimuli [145], or a lower number of connexins is present or redistributed from intercalated disks to lateral cell borders in several cardiac diseases, creating an arrhythmogenic “gap junction remodelling” [146]. In cancer, connexins appear to have a contrasting role: while in some cases they promote tumor suppression, in others, they enable the growth and invasiveness of cancer cells through cell communication and the microenvironment [147]. Some studies have shown that increased expression of connexins can generate tumors with more aggressive characteristics [148,149].

The overexpression of Panx1 has been correlated with poor prognosis in multiple cancers [150,151], especially in pancreatic adenocarcinoma (PAAD), and immune infiltration of macrophages, neutrophils, and fibroblasts [150]. An in vitro study in the 4T1 cell line suggested that tumors may induce neutrophil extracellular traps (NETs) by the release of spermidine via Panx1: Panx1-deficient 4T1 cells showed a reduction in tumor volume to control cells in vivo when subcutaneously transplanted into mice, whereas the inhibition of spermidine synthesis suppressed tumor growth in the mouse transplant model [152]. The implication of NET release by tumor-associated neutrophils (TANs) was also verified by transplanting wild-type 4T1 tumor cells into Padi4^−/−^ mice, which resulted in the reduction in tumor size. PBN also reduced tumor size in the colon [151]. Patients with colon cancer had increased levels of Panx1 mRNAs, but there were no differences depending on the stage of the cancer. However, there was a higher abundance of Panx1 in tumor cells and stroma from patients with tumor–node–metastases (TNM) III compared to patients with TNM I and II. Panx1 expression was also more abundant in patients with cancer on the right side of the colon compared to those who had it on the left side, suggesting the existence of molecular subtypes in cancer of the right colon.

However, Panx1-deficient mice with induced melanoma (BPC) showed no differences in primary tumor formation or survival compared to BPC-Panx1^+/+^ mice; on the other hand, BPC-Panx1^−/−^ mice had a higher infiltration of CD8+ T lymphocytes in the TME [153]. Panx1 has also been correlated with poor prognosis in breast cancer [154]. Panx1 expression was positively correlated with higher extracellular ATP and adenosine levels in the TME, which induced an immunosuppression mechanism also supported by high expression of CD39/CD73, due to the tumor-associated neutrophil increase. In the metastatic breast cancer line, it was found that the truncated Panx^11_89^ protein acted as an activator of Panx1, inducing ATP release from cells and promoting breast cancer cell survival during traumatic intravascular deformation through purinergic receptors P2Y [155]. In colorectal cancer, TNFα was found to sustain cancer immunogenicity by promoting the release of ATP via purinergic receptor P2X7 (P2X7R) [156].

Panx1 was found to be highly abundant also in invasive pituitary adenoma (PA) compared with noninvasive PA and the pituitary gland. In the GH3 mouse cell line, the overexpression of Panx1 promoted cell proliferation, while the probenecid (PBN, pannexins blocker) reversed the effect. Overexpression of Panx1 also led to alteration in different metabolic pathways such as glycolysis, and amino acid and lipid metabolism, with altered expression of genes like arginase 2 (ARG2), glutaminase, and indoleamine-2,3-dioxygenase-1 (IDO1). In GH3 cells, Panx1 promoted the invasion by increasing ATP release and causing the activation of P2X7R, which then triggered Ca^2+^ influx and further impacted gene and protein expression of MMP2/9 [157]. However, the effect was reversed by PBN, ATPase, JNJ (P2X7R specific antagonist), and nifedipine (L-type calcium channel inhibitor).

Metastatic melanomas showed the loss of GJA1 (Cx43) protein, the increase in cytoplasmic GJB2 (Cx26), and the upregulation of both GJC3 (Cx30.2) and GJB1 (Cx32) through melanoma progression, compared to melanocytes. The same study also revealed a bidirectional interaction between cancer and the microenvironment, with an increase in Cx43, Cx26, and GJB6 (Cx30) proteins in the adjacent epidermis, and in Cx43 in lymphoid vessels close to the tumor [158]. In osteocytes, the activation of Cx43 suppressed breast cancer cell migration, invasion, and growth [159] by increasing the release of ATP, which triggered P2X7R signaling in breast cancer cells. The treatment with Cx43-M2 antibodies anti-Cx43 induced the opening of Cx43 hemichannel in osteocytes, inhibiting cancer cell growth and migration in mouse breast carcinoma, decreasing the levels of immune-suppressive Treg cells and increasing tumor-infiltrating T cells and Th lymphocytes [160]. The biphasic effect of ATP is due to a dose-dependent behavior, which promotes inhibition at a lower dosage and the stimulation at higher dosage, as already observed on breast cancer cells [161]. In colorectal cancer cell lines, the overexpression of Cx43 inhibited cell migration and invasion, whereas its downregulation supported the stemness of cells by reducing the cell stiffness, thus reducing drug permeability and enhancing cell aggressiveness caused by drug resistance [162].

Extracellular vesicles released by glioblastoma cancer cells boosted tumor invasiveness through Cx43, which acted by modulation of calcium signaling and formation of Cx43-mediated connections [163]. Furthermore, the pharmacological treatment of gap junctions with carbenoxolone strongly inhibited the spontaneous Ca^2+^ oscillation of non-periodic cells in glioblastoma, resulting in reduced network communication in glioma [164].

GJA5 (Cx40) participates in both the proliferation and migration of the extravillous trophoblast Jeg-3 cell line in hypoxia: indeed, cells expressing low levels of Cx40 displayed a more migratory phenotype, whereas cells with high levels of Cx40 exhibited a more proliferative phenotype. Hypoxia also promoted the formation of Cx40-associated plaques between neighboring cells, a response mimicked by the activation of the nitric oxide/guanosine-3′,5′-cyclic monophosphate/protein kinase cGMP-dependent 1 (NO/cGMP/PKG) pathway [165].

GJB3 (Cx31) expression was highest in head and neck squamous cell carcinoma (HNSC) and lowest in uveal melanoma (UVM). GJB3 was upregulated in several cancers, and it was linked to shorter overall survival (OS) in some of them, while in other cases a higher expression of GBJ3 was correlated to a longer OS; this differential result suggested both tumor-suppressive and tumor-promoting properties of the hemichannel, mainly depending on the type of cancer. Furthermore, GJB3 knockdown reduced lung cancer cell proliferation and migration, inhibited the PI3K/AKT pathway in lung adenocarcinoma (LUAD), pancreatic adenocarcinoma (PAAD), and mesothelioma (MESO), and altered pathway activity in H2030, PANC1, and H2452 cancer cell lines [166].

The overexpression of miR-130a/b can directly reduce Cx43 and affect intercellular communications of bone marrow mesenchymal stromal cells (BMSCs), whilst the high Cx43 expression could reverse the effect, enhancing adipogenesis and restoring the osteogenic differentiation capacity of BMSCs [167].

**Table 1:** Effects of ion channels in cancer. The table highlights the roles of K_ATP_, TRPM5, connexins, and pannexins in different types of cancer, their effects on tumor progression, and corresponding references. The upwards arrow (↑) indicates promotion of tumor growth, whereas the downwards arrow (↓) refers to the suppression.

Ion Channel	Tumor Growth Promotion (↑) or Suppression (↓)	Other Effects	References
**K_ATP_**	Liver cancer ↑↓	Maintenance of membrane potential and survival	[79]
	Glioblastoma ↑	Increased Ca^2+^ influx	[80,81]
	Pancreatic cancer ↑	Activation of growth and survival pathways	[86]
	Gastric cancer ↑	Adaptation to hypoxia and acidic TME	[82,95,96]
	Bladder cancer ↑↓	Increased metabolic resilience	[88,89]
	Prostatic cancer ↑	Role in viral tumorigenesis	[87]
	Cervical cancer ↑	Interaction with lipid metabolism	[97,98]
	Colorectal cancer ↓	Potential therapeutic target	[84]
**TRPM5**	Colon adenocarcinoma ↑ Breast cancer ↑ Lung cancer ↑ Gastric cancer ↑ Colorectal cancer ↑ Melanoma ↑	Drug resistance Independent prognostic value Correlation with worse prognosis or prognostic significance Increased expression in aggressive tumor cells Blockade reduces lung metastasis Association with shorter survival in certain cancer Cellular adaptation to low pH conditions	[127] [128] [129] [130] [130] [130] [130,131]
**Pannexins** **Connexins**	Pancreatic adenocarcinoma ↑ Breast cancer ↑ Melanoma ↑ Colorectal cancer ↑ Pituitary adenoma ↑ Glioblastoma ↑ Breast cancer ↓ Colorectal cancer ↓ Melanoma ↑↓ Lung cancer ↓ Pancreatic cancer ↓ Mesothelioma ↑ Head and neck squamous cell carcinoma ↑	Panx1 overexpression correlates with poor prognosis in multiple cancers Panx1 promotes tumor growth, invasion, and immune evasion Panx1 deficiency reduces tumor size and increases CD8+ T cell infiltration Panx1 enhances immunosuppression Panx1 contributes to metabolic reprogramming Cx43 loss is linked to metastasis Cx43 suppresses tumor growth and invasion Cx43 enhances invasiveness via extracellular vesicle signaling Cx43 inhibition disrupts tumor cell communication and reduces network connectivity Cx40 regulates migration and proliferation in hypoxic tumor cells Cx31 has variable effects, promoting or suppressing tumors depending on cancer type	[150,151,154] [150,157] [153] [154] [157] [158] [159,162] [163] [164] [165] [166]

## 3. Discussion

The establishment of a bidirectional interaction between tumor cells and the surrounding microenvironment is needed to support the growth of tumor cells themselves, although detrimental to healthy cells. Because many other ion channels are also involved in cancer progression, it is crucial to understand the mechanisms and alterations in the context of the tumor microenvironment to adopt the best therapeutic strategy to treat cancer. Higher lactate concentration, as well as ATP production and calcium signaling, may affect the activity of the ion channels presented here, disrupting the homeostasis of cells and promoting the development of cancer. Recently, the role of bitter taste receptors (TAS2Rs) has been discussed [168]. Natural bitter compounds, which are generally introduced in the body with the diet and metabolized, can reach the TME and bind TAS2Rs [169,170], activating a signaling cascade which induces the increase in intracellular Ca^2+^ and the activation of TRPM5; at the same time, the signaling cascade induces the activation of large-pore channel pannexins, which can release in the TME molecules of ATP [171], creating a loop of events. As already reported above (see Section 2.3), cancer cells may also affect the healthy stromal cells in the surroundings, by communicating via cell–cell gap junctions and pannexins, by releasing oncometabolites [165,167]. In fact, the TME represents the playground of cancer cells, which owe their fame to their ability to adapt in unstable and demanding conditions, such as hypoxia and mild acidity, rapidly emerging and proliferating. Thus, after genetic and epigenetic changes, cancer cells survive and eventually metastasize [172]. The acidosis typical of the TME was found to support the formation of metastasis [173]. In this context, the treatment with TPPO, a selective TRPM5 blocker, significantly inhibited the formation of lung metastasis. Furthermore, the overexpression of Cx43 was proved to reduce lung metastasis, both in terms of size and number, in a mouse xenograft model [174]. These effects may be due to their impact on the epithelial-to-mesenchymal transition (EMT), an important step of the tumor progression that is usually associated with a higher risk of metastasis development [175].

Recent single-cell and spatial transcriptomic analyses have significantly advanced our understanding of ion channel regulation in cancer. The study by He et al. highlighted the impact of oxidative stress on glioma progression, showing that AP-1 transcription factors mediate stress responses that influence tumor cell survival [176]. Additionally, EMT-related pathways are upregulated, potentially promoting glioma invasiveness and therapy resistance. Meanwhile, the review by Jin et al. [177] discussed how spatial transcriptomics revealed tumor heterogeneity and microenvironmental interactions, including the differential expression of ion channels across tumor regions. This approach enabled researchers to map functional states of cancer cells in situ, providing insights into how local signaling and metabolic conditions regulate ion transport and contribute to malignancy. These findings emphasized the importance of integrating single-cell and spatial approaches to uncover the spatiotemporal regulation of ion channels, which could lead to novel therapeutic targets in cancer.

Epigenetic mechanisms can occur in cancer and after exposition to the tumor microenvironment. The exposure to a transient extracellular acidification slightly altered the mRNA expression of Trpm5 in the LLCm1 lung cancer cell line, differently from chronic exposure that induced a high expression of the channel [131]. Cx genes are regulated by DNA methylation in tumor progression, too [178]. In the inflammatory microenvironment, pro-inflammatory mediators induced the reduction in Cx43 expression; additionally, pre-tumorigenic cells which had lost Cx43 were susceptible to the inflammatory microenvironment and acquired a motile phenotype [179]. Cx43 silencing in HeLa cells and hypermethylation of Cx32 and Cx45 have been linked to reduced expression in several cancers [180].

The therapeutic targeting of ion channels represents an innovative approach in oncology, aimed at modulating fundamental cellular processes such as proliferation, apoptosis, migration, and metabolism while selectively targeting cancer cells [181]. TRPM5, involved in intracellular calcium signaling and metabolic regulation, is often overexpressed in cancer cells, promoting their survival and proliferation. To inhibit its activity, compounds such as diphenylphosphoryl azide and triphenylphosphine derivatives are studied, as they interfere with the function of the channel and induce apoptosis [182]. Additionally, nanoparticles have been developed for the targeted delivery of these inhibitors, increasing their effectiveness and reducing systemic side effects. The use of nanoparticles for the selective delivery of ion channel inhibitors represents further innovation, optimizing drug bioavailability and minimizing systemic adverse effects [183].

K_ATP_ channel inhibitors, originally developed for diabetes, are emerging as potential cancer therapies. Glyburide reduced tumor proliferation in bladder and lung carcinoma by modulating the cell cycle and inhibiting the NLR family pyrin domain containing 3 (NLRP3) inflammasome [184]. In prostate cancer, it induced Ca^2+^-independent cell death [185]. Glipizide, in combination with atrial natriuretic peptide, hinders breast cancer growth and angiogenesis [186]. Additionally, it enhanced apoptosis in lung adenocarcinoma cells [187] and reduced microvascular density in prostate cancer [188]. The glimepiride–metformin adduct (GMA) enhanced cell cycle arrest and apoptosis in breast cancer [189]. Nitrated nateglinide (NO_2_-NAT) induced apoptosis in pancreatic cancer [190], while repaglinide inhibited forkhead box O3 (FOXO3), reducing neuroblastoma cell migration [191]. Among the activators, pinacidil induces apoptosis in HepG2 hepatoblastoma cells [192], while cromakalim inhibits neuroblastoma and astrocytoma cell growth, with its effects reversed by sulfonylureas [193]. Additionally, cromakalim has been found to stimulate DNA synthesis in liver cells, suggesting potential applications in liver cancer therapy [83]. Diazoxide has been shown to stimulate protein accumulation in bladder carcinoma cells without increasing proliferation, indicating a complex role in cancer cell growth [194]. Minoxidil exhibited diverse effects: it promoted SUR2A subunit expression in renal and canine breast tumors [85], reduced breast cancer cell invasion, particularly when combined with ranolazine, and increased blood–tumor barrier permeability in brain tumors, potentially enhancing drug delivery [195]. 

Pannexins and connexins can regulate tumor growth and response to treatments. Modulating these channels can limit cancer cell proliferation and enhance chemotherapy effectiveness [180]. Pannexins, particularly Panx1, also have a significant impact on tumor progression. This protein is activated during apoptosis and facilitates ATP release, a signaling molecule that stimulates the immune response against tumors [196]. Some studies have indicated that drugs like ivermectin can activate Panx1 and induce cancer cell death, as observed in breast carcinoma [197]. Additionally, Panx3 may serve as a useful biomarker for certain tumor types, while Panx1 mutations have been linked to metastatic breast cancer [198]. Beyond their role in tumor growth, pannexins also play a role in managing chemotherapy-induced pain. Panx1, together with the P2X7R receptor, has been identified as a key element in neuropathic pain caused by drugs like oxaliplatin. Consequently, Panx1 inhibitors, such as Panx10 or erioglaucine, could provide an effective strategy for reducing chemotherapy side effects without compromising its efficacy [13,199]. Various natural and pharmacological compounds can influence connexins, too. Substances such as lycopene, vitamin D, carotenoids, and statins have been shown to enhance communication through GJIC, thereby contributing to tumor growth reduction [200,201]. Several strategies have also been developed to directly modulate connexins activity. The use of mimetic peptides such as αCT1 and TAT-Cx43 has been shown to reduce tumor growth and increase the sensitivity of cancer cells to chemotherapy [202,203]. Monoclonal anti-Cx43 antibodies were proven to be particularly useful in improving drug distribution in brain tumors [204], while siRNA-mediated inhibition of Cx37 has been associated with increased apoptosis rates in gastric cancer cells [205]. However, the role of Cx43 varies depending on the type of cancer: in lung cancer, it appeared to prevent cisplatin resistance, whereas in glioblastoma, it may contribute to temozolomide resistance [206,207]. To enhance the effectiveness of cancer therapies and reduce drug resistance, combinatory approaches integrating various inhibitors are being developed, leading to enhanced therapeutic effects [208]. At the same time, advancements in precision medicine are driving increasingly personalized treatments, based on identifying the tumor’s molecular profile and selecting the most appropriate therapy for each patient [209].

Regrettably, the study of ion channels within the TME still presents some limitations. The complexity and heterogeneity of the TME make it difficult to accurately study the function of ion channels in vivo and current experimental models often do not faithfully replicate the physiological condition, which can be a limitation for the translation of the results into the clinical setting. Moreover, in some cases, the ion channels mentioned above showed both pro-apoptotic and anti-apoptotic roles, which make it challenging to develop a targeted and universally applicable therapeutic strategy.

## 4. Conclusions and Perspective

This review has investigated the role of ion channels, in particular K_ATP_, TRPM5, and gap junctions, as connexins and pannexins within the tumor microenvironment, which play a crucial role in cancer progression and cell communication. From a therapeutic point of view, although some ion channels have been proposed as targets, effective and specific strategies are still lacking. For this reason, novel therapeutic approaches targeting specific ion channels with selective blockers or modulators, like in the case of TPP-based conjugates or compounds that act as activators or inhibitors of tumor cell growth, along with the use of mimetic peptides that influence sensitivity of cancer cells to chemotherapy, could pave the way for new opportunities in cancer therapy.
